# Predictive value of the random forest model based on bioelectrical impedance analysis parameter trajectories for short-term prognosis in stroke patients

**DOI:** 10.1186/s40001-024-01964-8

**Published:** 2024-07-24

**Authors:** Jiajia Yang, Jingjing Peng, Guangwei Liu, Feng Li

**Affiliations:** 1 Department of Neurology, The First Affiliated Hospital of Chongqing Medical University, Chongqing, 400016 People’s Republic of China; 2 Department of Neurosurgery, The First Affiliated Hospital of Chongqing Medical University, Chongqing, 400016 People’s Republic of China

## Abstract

**Background:**

The short-term prognosis of stroke patients is mainly influenced by the severity of the primary disease at admission and the trend of disease development during the acute phase (1–7 days after admission).

**Objective:**

The aim of this study is to explore the relationship between the bioelectrical impedance analysis (BIA) parameter trajectories during the acute phase of stroke patients and their short-term prognosis, and to investigate the predictive value of the prediction model constructed using BIA parameter trajectories and clinical indicators at admission for short-term prognosis in stroke patients.

**Methods:**

A total of 162 stroke patients were prospectively enrolled, and their clinical indicators at admission and BIA parameters during the first 1–7 days of admission were collected. A Group-Based Trajectory Model (GBTM) was employed to identify different subgroups of longitudinal trajectories of BIA parameters during the first 1–7 days of admission in stroke patients. The random forest algorithm was applied to screen BIA parameter trajectories and clinical indicators with predictive value, construct prediction models, and perform model comparisons. The outcome measure was the Modified Rankin Scale (mRS) score at discharge.

**Results:**

PA in BIA parameters can be divided into four separate trajectory groups. The incidence of poor prognosis (mRS: 4–6) at discharge was significantly higher in the “Low PA Rapid Decline Group” (85.0%) than in the “High PA Stable Group “ (33.3%) and in the “Medium PA Slow Decline Group “(29.5%) (all *P* < 0.05). In-hospital mortality was the highest in the “Low PA Rapid Decline Group” (60%) compared with the remaining trajectory groups (*P* < 0.05). Compared with the prediction model with only clinical indicators (Model 1), the prediction model with PA trajectories (Model 2) demonstrated higher predictive accuracy and efficacy. The area under the receiver operating characteristic curve (AUC) of Model 2 was 0.909 [95% CI 0.863, 0.956], integrated discrimination improvement index (IDI), 0.035 (*P* < 0.001), and net reclassification improvement (NRI), 0.175 (*P* = 0.031).

**Conclusion:**

PA trajectories during the first 1–7 days of admission are associated with the short-term prognosis of stroke patients. PA trajectories have additional value in predicting the short-term prognosis of stroke patients.

**Supplementary Information:**

The online version contains supplementary material available at 10.1186/s40001-024-01964-8.

## Introduction

Stroke is the second leading cause of death and a major contributor to long-term disabilities worldwide. Exploring the influencing factors associated with poor prognosis is of great significance for diagnosis, treatment and improvement of the prognosis of stroke patients [1]. Post-stroke brain injury mainly includes primary brain injury and secondary brain injury [2]. Primary brain injury is irreversible neuronal damage that occurs immediately after stroke due to tissue destruction caused by severe ischemia or hematoma. In contrast, secondary brain injury is reversible neuronal damage caused by ischemia and hypoxia changes in brain tissue due to factors such as excitotoxicity, oxidative stress and inflammation that occur after the primary brain injury, the most severe consequence of which is progressive brain edema, leading to neuronal cell death, exacerbating primary brain injury, and causing systemic dysfunction in other organs, ultimately resulting in poor prognosis [3, 4]. Therefore, in addition to primary brain injury, secondary brain injury after stroke also plays an important role in the poor prognosis of this group of patients [5]. Among them, the dynamic changes of the inflammatory response and nutritional status during the acute phase (1–7 days after stroke onset [6]) are critical in the mechanism of secondary brain injury and disease progression [7–11]. During the acute phase of brain injury, Damage-Associated Molecular Patterns (DAMPs) released from damaged cells can trigger local and systemic inflammatory responses, exacerbating post-stroke brain edema and vasospasm [2, 12], leading to the development of delayed brain injury and systemic secondary complications (such as stroke-associated pneumonia [13], gastrointestinal dysfunction [14], and cardiorenal insufficiency [15, 16]), severely affecting clinical outcomes in this group of patients. In addition, the inflammatory response during the acute phase in first 1–7 days of admission can cause increased catabolism, resulting in greater nutritional demands. Coupled with factors such as impaired consciousness, neurogenic swallowing disorders and gastrointestinal dysfunction, nutritional intake becomes insufficient, further deteriorating the nutritional status in this group of patients. The persistence and progressive development of this hypoimmune hypermetabolic state further aggravates inflammation, creating a vicious circle that leads to a poor prognosis and even accelerates mortality in stroke patients [17–19]. Therefore, the early prognostic evaluation of stroke patients should integrate the severity of the primary disease injury at admission and the changes in inflammation and nutritional status during the acute phase.

Currently, the indicators that can be used to predict the prognosis of stroke patients include National Institutes of Health Stroke Scale (NIHSS), Red Blood Cell Distribution Width (RDW), Prognostic Nutritional Index (PNI), and Hemoglobin, Albumin, Lymphocyte, and Platelet score (HALP) [20–23]. However, NIHSS mainly reflects the degree of primary neurological impairment at the onset of disease and cannot assess the dynamic course and trend of acute-phase inflammation and nutritional status along with the pathophysiological development. Previous studies have indicated that the main indicators available to assess inflammation and nutritional status are biochemical indicators, including RDW, PNI and HALP [22–24]. However, the detection of biochemical indicators is usually invasive and limited by disease diagnosis and treatment, making it inconvenient for dynamic assessment. Therefore, there is a need to explore clinically valid indicators for the dynamic assessment of inflammation and nutritional status during the acute phase of stroke patients, which also have predictive value for their prognosis. Bioelectrical impedance analysis (BIA) has now been widely used in the assessment of inflammation and nutritional status and prognosis prediction in patients with cancer, hemodialysis and heart failure because of its objective, noninvasive, easy-to-use, reproducible and rapid results [25–28]. Among the BIA parameters, Phase Angle (PA), Skeletal Muscle Mass (SMM) and edema index (ECW/TBW) are considered to be closely related to inflammation and nutritional status [29–32]. Among them, PA is the most classic and most concerned effective prognostic indicator [33]. It is the ratio of reactance (Xc) to resistance (R) obtained by BIA measurement and expressed as an Angle that can be used to assess membrane integrity and cell function, with lower PA associated with damage to cell structure and increased cell death [34]. However, there is limited research on BIA in stroke patients. Further validation is needed to continue exploring the value of prognostic prediction in this group of patients by examining its dynamic changes.

This study prospectively collected clinical data, inflammation and nutrition-related biochemical indicators at admission, as well as BIA parameters during the first 1–7 days of admission from 162 stroke patients. A Group-Based Trajectory Model (GBTM) was constructed for the BIA parameters, and the random forest algorithm was utilized to select BIA parameters and clinical indicators with predictive value, to clarify the relationship between the dynamic changing trajectory of BIA parameters and clinical outcomes in this group of patients and to construct a predictive model for the short-term prognosis of stroke patients. This study seeks to identify a non-invasive and continuously observable bedside assessment tool for clinical observation of stroke patients during the acute phase, especially the early identification and dynamic changes in inflammation and nutritional status, providing reference for early comprehensive and accurate prognostic prediction for these patients.

## Methods

### Study design and participants

This is an observational study that includes stroke patients admitted to the Department of Neurology, the First Affiliated Hospital of Chongqing Medical University, from July 2022 to January 2023. Continuous BIA measurements were performed within the first 1–7 days of admission in patients who met the inclusion criteria, and clinical data were prospectively collected throughout their hospital stay. The outcome was grouped by mRS scores at discharge and analyzed. The study was conducted in accordance with the Declaration of Helsinki, approved by the Medical Ethics Committee of the First Affiliated Hospital of Chongqing Medical University, and registered in the Chinese Clinical Trials Registry (www.chictr.org.cn/) under the registration number ChiCTR2300069198.

#### Inclusion criteria

1. Age ≥ 18 years;
2. A brain computed tomography (CT) scan or magnetic resonance imaging (MRI) scan was performed before or during hospitalization, meeting the World Health Organization (WHO) diagnostic criteria for stroke [35], with a confirmed diagnosis of either ischemic or hemorrhagic stroke;
3. Patients in the acute phase of stroke, i.e., within 1–7 days of symptom onset [6].
4. All patients or their guardians provided written informed consent.

#### Exclusion criteria

**Fig. 1:**
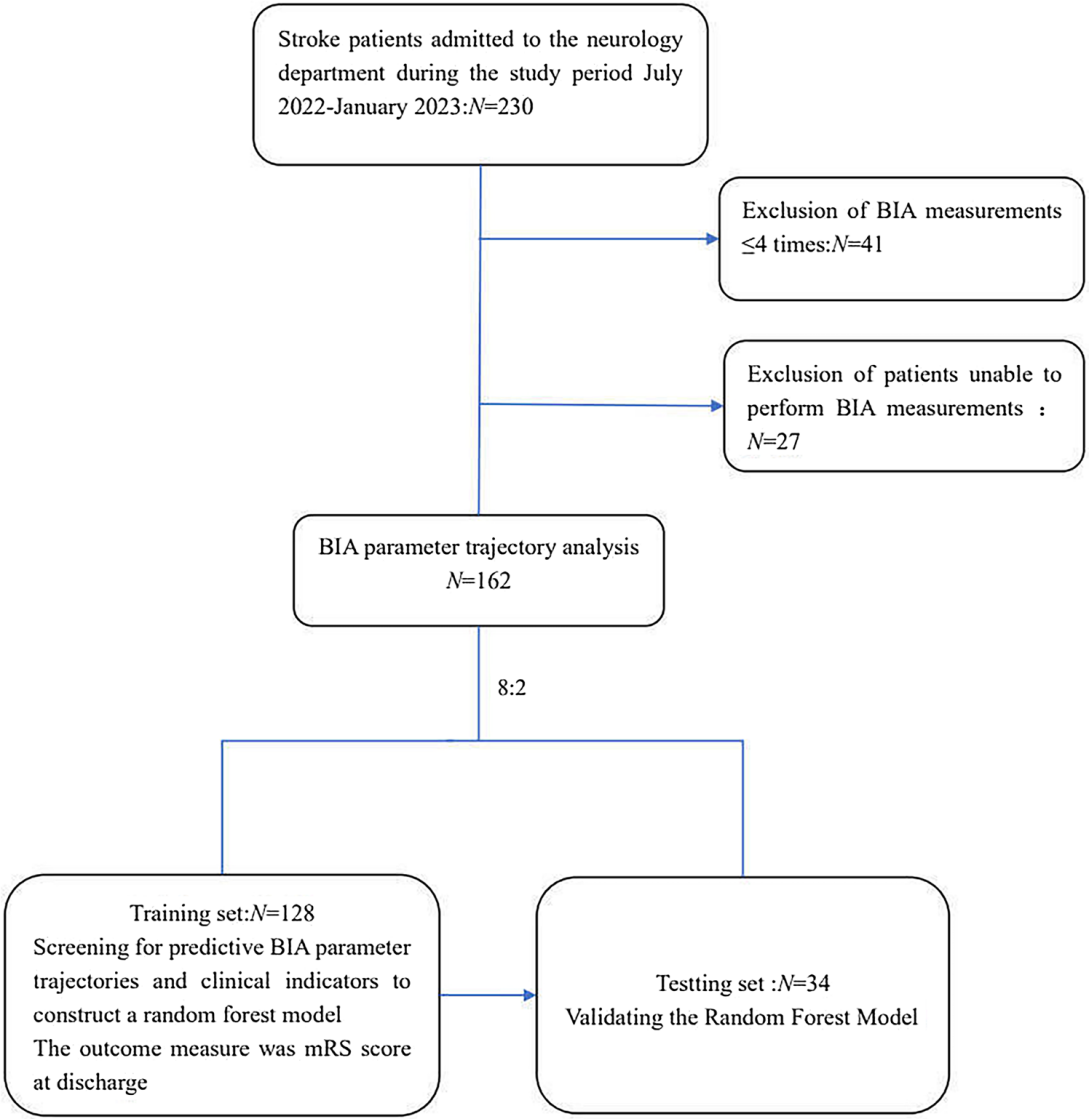
Patient recruitment flowchart. *BIA* bioelectrical impedance analysis, *mRS* the Modified Rankin Scale

1. Patients who are unable to undergo BIA measurements due to hemodynamic instability, amputation, implantation of a metallic device (such as a pacemaker or artificial femoral head), skin damage in the area where the BIA electrodes are attached, etc.;
2. BIA continuous measurement times ≦ 4 times (see Fig. 1).

### Outcome indicators

Primary outcome indicator: Modified Rankin Scale (mRS) score at discharge [36]. The mRS is scored from 0 to 6, where 0 indicates no symptoms; 1 indicates symptoms but no impact on daily activities and work; 2 indicates slight disability, where some activities of daily living cannot be performed but can manage daily tasks without assistance; 3 indicates moderate disability, requiring some assistance but can walk without assistance; 4 indicates moderately severe disability, where the patients is unable to walk and meet self-care needs without assistance; 5 indicates severe disability, with complete dependence on others for daily living; 6 indicates death. Higher scores indicate more severe neurological impairment. An mRS score of 0–3 represents a favorable neurological prognosis, while a score of 4–6 represents a poor neurological prognosis.

### Sample size calculation

This research adopted the sample size estimation for prediction model recommended by Richard D. Riley [37]. According to previous literature, the rates of poor prognosis in stroke patients at hospital discharge ranged from 33.2 to 55.1% [38, 39], and 44% was taken in this study. Assuming that the number of predictors is between 5 and 7, and the root mean square error (Criterion value rMPSE) is 0.11–0.12, at least 160 patients are required for this research.

### Data collection methods

1. Clinical data: including age, gender, day 1 admission-related indicators body mass index (BMI), National Institutes of Health Stroke Scale (NIHSS), Charlson Comorbidity Index (CCI) and diagnosis (Ischemic stroke and hemorrhagic stroke);
2. Biochemical indicators within the first day of admission: including albumin (Alb); total lymphocyte count (TLC); RDW; PNI; Hemoglobin, Albumin, Lymphocyte and Platelet Score (HALP);
3. BIA parameters from 1 to 7 days after admission: including PA, SMM, fat-free mass (FFM), body cell mass (BCM), extracellular mass/body cell mass (ECM/BCM) and extracellular water/total body water (ECW/TBW).

All clinical data were recorded and collected by 2 dedicated research assistants through an electronic medical record system. All BIA data were measured and recorded by 2 dedicated dietitians using the BIA (InBody S10) instrument (see Appendix for detail), and data entry was performed separately. Following data entry, the patient’s name and medical record number were removed and the patient was given a unique study number. A designated study coordinator organized and confirmed the accuracy of all data, and manually verified inconsistent or out-of-range values. The data set was validated and cleaned prior to statistical analysis to prevent any further changes and to ensure consistency and integrity of statistical reporting and analysis data. All researchers who collected and collated the data were unaware of the study.

### Definition of clinical and laboratory indicators

Relevant clinical indicators and definitions of BIA parameters in the study are presented in the appendix (please refer to the Additional file 1 for details).

### Trajectory group modeling

The trajectory analysis model is mainly used for tracking data analysis of the aggregate with heterogeneity. Its principle is to explore the subgroups with different development trajectories in the aggregate for the existence of heterogeneity in the aggregate, and then analyze the development trends of different subgroups and their trajectory characteristics. In this study, we applied the group-based modeling of longitudinal data using the software Stata15.1 (StataCrop LLC 4905 Lakeway Drive College Station TX77845 USA) to determine trajectories of BIA parameters in stroke patients during the first 1–7 days of admission. The number of subgroups of the BIA parametric trajectory model and the functional model of each subgroup are determined according to the following principles: the Bayesian Information Criterion (BIC) with the minimum absolute value and the Average Post-hoc Grouping Probability (AvePP) > 0.70 for each trajectory group. The closer the absolute value of BIC to 0, the better the model fits, and AvePP > 0.70 indicates a high degree of conformity between the members within the subgroup and their corresponding trajectory after grouping according to the trajectory [40].

### Prediction model construction and model comparison

Random forest (RF) is an integrated machine learning (ML) algorithm. Through the use of bagging technique, it has introduced random selection attributes during the training process based on decision trees. RF is characterized by its simplicity, easy implementation, and low computational cost, and it has demonstrated excellent performance in numerous real-world tasks, such as assisting in clinical decision-making and predicting prognosis [41]. In this study, the Boruta package in R (version 4.0.2, R Foundation for Statistical Computing, Vienna, Austria, https://www.R-project.org/) was used to select important characteristic variables with predictive value. The random forest package was employed to construct a prediction model for the functional outcome of stroke patients at hospital discharge. Internal validation of the model was performed using tenfold cross-validation. The AUC, accuracy, sensitivity, and specificity were applied to describe the predictive value of the model. The DeLong’s test, DCA, NRI, and IDI were used to describe the prediction performance of different models. These four methods were implemented using the pROC package, rmda package, nricens package, and PredictABE package package in the R language, respectively.

### Description of statistical methods

All data in this study were entered through (Access 2016) and analyzed for differences using SPSS (version 25.0). Normally distributed metric data were described as mean ± standard deviation (*x* ± *s*). Comparisons between groups were performed using independent samples *t* test (two groups) or ANOVA (three and more groups), with post-hoc pairwise comparisons using the SNK-*q* test. Skewed distributed metric data were expressed as median (interquartile range) (M(IQR)). Comparisons between groups were conducted using the Mann–Whitney *U* test (two groups) and the Kruskal–Wallis test (three and more groups), with pairwise comparisons adjusting P values using the Dunn–Bonferroni method. Categorical variables were expressed as counts and percentages. Comparisons between groups were carried out using the chi-square test, with post-hoc pairwise comparisons adjusting *P* values using the Bonferroni method. A *P* value < 0.05 was considered statistically significant.

## Results

### Analysis of the general data of two groups of patients with good and poor prognosis

During the study period, a total of 230 stroke patients were admitted to the Neurology Department. Among them, 68 patients were excluded for reasons such as inability to measure BIA. Finally, 162 patients were included for analysis. Table 1 shows the comparison of the clinical data and biochemical indicators between the two groups of patients. In the clinical data, the poor prognosis group had higher age, NIHSS and CCI at admission and lower BMI compared to the good prognosis group; in biochemical indicators, compared to the good prognosis group, the poor prognosis group had lower Alb, TLC, PNI, and HALP and higher RDW. The differences between the two groups were statistically significant (*P* < 0.05), whereas the differences in gender and diagnosis were not statistically significant between the two groups (*P* > 0.05).

**Table 1:** Comparison of clinical data and the characteristics of biochemical indicators between two groups of stroke patients with different prognosis

Characteristics	Total	Group		*χ*^2^/*t*/*Z*	*P* value
		Good (mRS 0–3) (*n* = 92)	Poor (mRS 4–6) (*n* = 70)		
Clinical data					
Age (years)	69.0 [58.0–76.0]	67.5 [58.0–74.0]	72.5 [64.0–80.0]	9.035	0.003
BMI	24.2 ± 3.2	24.9 ± 2.8	23.2 ± 3.4	12.650	< 0.001
Sex					
Male	105 (64.8)	60 (65.2)	45 (64.3)	0.015	0.902
Female	57 (35.2)	32 (34.8)	25 (35.7)		
Diagnosis					
Ischemic stroke	131 (80.9)	77 (83.7)	54 (77.1)	1.103	0.294
Hemorrhagic stroke	31 (19.1)	15 (16.3)	16 (22.9)		
NIHSS	6.0 [3.0–15.0]	3.0 [1.0–6.0]	16.0 [10.0–22.0]	77.446	< 0.001
CCI	1.0 [0.0–1.0]	0.0 [0.0–1.0]	1.0 [0.0–2.0]	8.921	0.003
Biochemical indicators on day 1 of admission					
Alb (g/L)	41.0 [37.0–43.0]	41.0 [38.5–43.0]	38.5 [35.0–44.0]	5.412	0.020
TLC (10^9^/L)	1.3 [0.8–1.8]	1.6 [1.2–2.0]	1.1 [0.7–1.5]	22.941	< 0.001
RDW (%)	13.2 [12.5–13.9]	12.9 [12.4–13.5]	13.5 [12.8–14.4]	13.645	< 0.001
PNI (g/L + 10^9^/L)	47.3 [43.0–51.8]	49.5 [44.8–53.0]	44.7 [39.5–49.4]	16.108	< 0.001
HALP (g/L)^2^	34.5 [23.4–48.7]	39.7 [30.3–53.5]	23.7 [14.6–38.9]	20.293	< 0.001

*Alb* albumin, *BMI* body mass index, *CCI* Charlson Comorbidity Index, *HALP* Hemoglobin, Albumin, Lymphocyte and Platelet Score, *NIHSS* National Institutes of Health Stroke Scale, *PNI* Prognostic Nutritional Index Score, *RDW* red blood cell volume distribution width, *TLC* total lymphocyte count

### Grouping process for BIA parametric trajectory modeling

GBTM was applied to identify the trajectories of BIA parameters (PA, FFM, BCM SMM, ECM/BCM and ECW/TBW) within the first 1–7 days of admission. Model evaluation indicators and the distribution of patients within each group are shown in Table 2. The optimal trajectory models selected for further study were Group PA4, Group FFM4, Group BCM3, Group SMM3, Group ECM/BCM2, and Group ECW/TBW2, with all groups having AVEPP > 0.7 and Entropy > 0.8. The fit of the trajectory analysis models with different subgroups are detailed in Additional file 2 Table S1.

**Table 2:** Evaluation indicators related to the modeling of BIA parameter trajectories in stroke patients within 1–7 days of admission

BIA parameters	Number of subgroups	LL	BIC	AIC	Entropy	Participants per group, *N* (%)	AVEPP
PA (°)	4	− 779.78	− 817.67	− 790.78	0.94	C1 = 20 (12.35%) C2 = 45 (27.78%) C3 = 61 (37.65%) C4 = 36 (22.22%)	C1 = 0.99 C2 = 0.96 C3 = 0.98 C4 = 0.98
FMM (kg)	4	− 2464.99	− 2492.54	− 2472.99	0.97	C1 = 43 (26.54%) C2 = 37 (22.84%) C3 = 49 (30.25%) C4 = 33 (20.37%)	C1 = 0.99 C2 = 0.98 C3 = 0.97 C4 = 0.99
BCM (kg)	3	− 2195.15	− 2215.81	− 2201.15	0.96	C1 = 50 (30.86%) C2 = 70 (43.21%) C3 = 42 (25.93%)	C1 = 0.99 C2 = 0.98 C3 = 0.99
SMM (kg)	3	− 2108.56	− 2129.23	− 2114.56	0.96	C1 = 50 (30.86%) C2 = 70 (43.21%) C3 = 42 (25.93%)	C1 = 0.99 C2 = 0.98 C3 = 0.99
ECM/BCM	2	2426.02	2408.80	2421.02	0.94	C1 = 108 (66.67%) C2 = 54 (33.33%)	C1 = 0.99 C2 = 0.96
ECW/TBW	2	2946.96	2933.23	2942.96	0.92	C1 = 120 (74.07%) C2 = 42 (25.93%)	C1 = 0.98 C2 = 0.98

*AIC* Akaike information criterion, *AVEPP* average posterior probability, *BIC* Bayesian information criterion, *BCM* body cell mass, *ECM* extracellular mass, *ECW* extracellular water, *FFM* fat-free mass, *PA* phase angle, *SMM* skeletal muscle mass, *TBW* total body water

### Screening of BIA parameter trajectories and other variables with prognostic predictive value

**Fig. 2:**
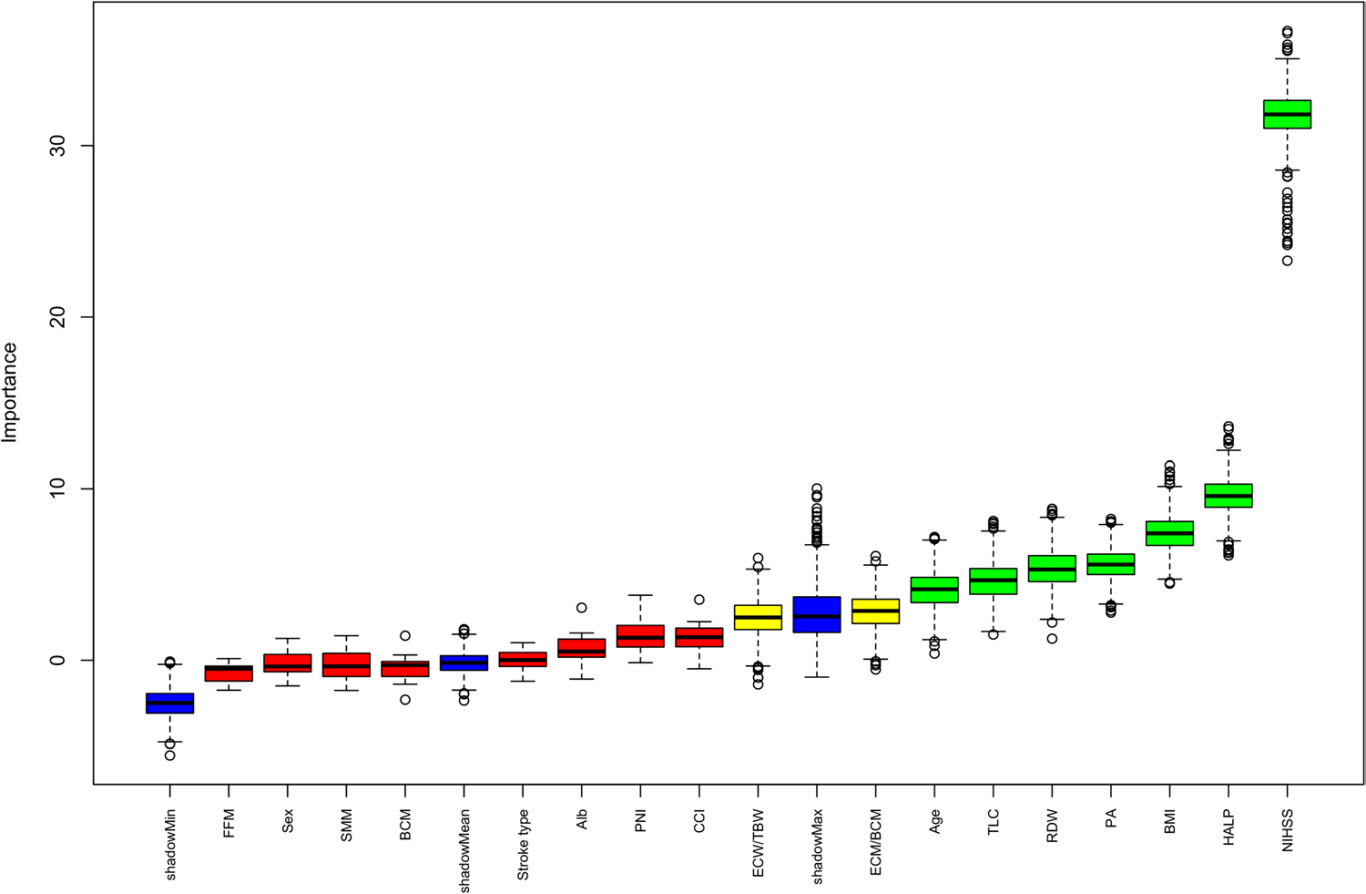
Screening of BIA parameter trajectories and other variables with prognostic predictive value. Box plots of all features attribute plus minimum, average, and maximum shadow scores; Green indicates feature variables with predictive value for prognosis, yellow indicates feature variables with unclear predictive value for prognosis, and red indicates feature variables with no predictive value. *Alb* albumin, *BMI* body mass index, *CCI* Charlson Comorbidity Index, *HALP* Hemoglobin, Albumin, Lymphocyte and Platelet Score, *NIHSS* National Institutes of Health Stroke Scale, *PNI* Prognostic Nutritional Index Score, *RDW* red blood cell volume distribution width, *TLC* total lymphocyte count, *BCM* body cell mass, *ECM* extracellular mass, *ECW* extracellular water, *FFM* fat-free mass, *PA* phase angle, *SMM* skeletal muscle mass, *TBW* total body water

A total of 17 parameters, all that indicators relevant to the BIA parameter trajectories (PA, FFM, BCM, SMM, ECM/BCM, ECW/TBW) and baseline characteristics analysis, were included and screened by applying Boruta algorithm. Ultimately, 7 parameters were identified to have predictive significance for poor prognosis at discharge (mRS: 4–6). Among all BIA parameter trajectories, PA trajectories demonstrated prognostic significance (see Fig. 2).

### Trend analysis of PA trajectories diagram and groups of trajectories

**Fig. 3:**
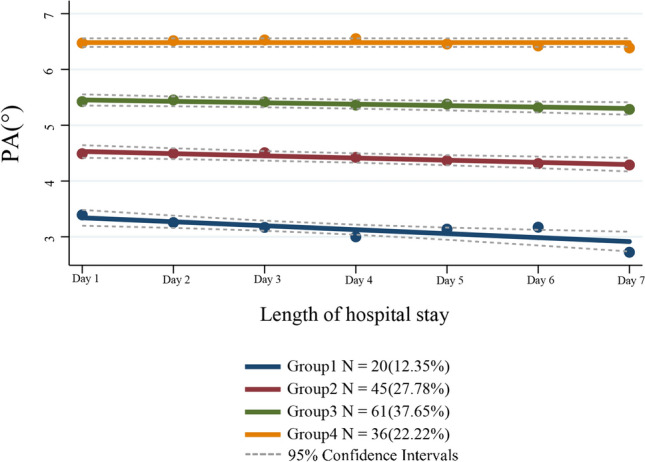
PA trajectory patterns within the first 1–7 days of admission. Trajectory models identified 4 distinct trajectory subgroups of PA in the stroke patients cohort. Solid lines show the mean PA levels for specific categories as a function of hospital stay. Dashed lines indicate estimated 95% confidence intervals. Orange, green, red and blue indicate the different trajectory groups respectively. *PA* phase angle

PA trajectories analysis showed that the initial mean values (on the first day of admission) for the PA trajectory groups were as follows: Group 1 (3.412), Group 2 (4.569), Group 3 (5.479), and Group 4 (6.481). Among them, the PA values in Group 1, Group 2, and Group 3 declined gradually over time, while there was no significant change in Group 4. Group 1 had the fastest decline rate in PA value (*β* = − 0.071,* P* < 0.05) compared to Group 2 (*β* = − 0.039, *P* < 0.05) and Group 3 (*β* = − 0.026, *P* < 0.05), (see Additional file 2 Table S2). Therefore, based on the initial value and decline rate of each trajectory group, the Group 1, Group 2, Group 3 and Group 4 were named as “Low PA Rapid Decline Group”, “Low PA Slow Decline Group”, “Medium PA Slow Decline Group”, and “High PA Stable Group” respectively. Each group of patients accounted for 12.35%, 27.78%, 37.65%, and 22.22% of the total number of patients, respectively (see Fig. 3).

### Characteristics analysis of the PA trajectory groups

The characteristic analysis of the four PA trajectory groups showed significant differences (*P* < 0.05) in clinical data (age, sex, NIHSS, CCI), biochemical indicators (Alb, TLC, PNI, RDW, HALP) and prognostic indicators (mRS at discharge, in-hospital mortality, length of hospital stay, total hospitalization cost, daily hospitalization cost) between the groups. Among them, the comparison of the main outcome indicator (mRS at discharge) showed that the incidence of poor prognosis (mRS: 4–6) at discharge was significantly higher in the “Low PA Rapid Decline Group” (85.0%) than in the “High PA Stable Group” (33.3%), *P* < 0.05. However, the NIHSS for the two group of patients at admission was (14.0 [9.5–22.0]) vs (7.5 [3.0–12.5]) respectively, and the total length of hospital stay was (11.5 [7.5–27.5] days) vs (16.0 [13.5–21.0] days), respectively, and the difference between those two groups was not statistically significant (*P* > 0.05) (see Table 3).

**Table 3:** Comparison of clinical data, the characteristics of biochemical indicators and prognosis among patients in different PA trajectory groups

Characteristics	PA trajectory groups				*χ*^2^/*F*	*P* value
	Group1 (*n* = 20)	Group2 (*n* = 45)	Group3 (*n* = 61)	Group4 (*n* = 40)		
Clinical data						
Age (years)	84.0 [76.5–89.0]	74.0 [69.0–80.0]	69.0 [59.0–73.0]^ab^	58.0 [49.0–64.5]^abc^	63.293	< .001
BMI	22.7 ± 3.7	23.4 ± 2.7	24.7 ± 3.1	25.0 ± 3.4	3.690	0.013
Sex					11.945	0.008
Male	8 (40.0)	26 (57.8)	41 (67.2)	30 (83.3)^a^		
Female	12 (60.0)	19 (42.2)	20 (32.8)	6 (16.7)^a^		
Diagnosis					10.314	0.016
Ischemic stroke	19 (95.0)	39 (86.7)	50 (82.0)	23 (63.9)		
Hemorrhagic stroke	1 (5.0)	6 (13.3)	11 (18.0)	13 (36.1)		
NIHSS	14.0 [9.5–22.0]	8.0 [3.0–18.5]	4.0 [2.0–10.0]^a^	7.5 [3.0–12.5]	14.3847	0.002
CCI	3.0 [1.0–6.5]	1.0 [0.0–2.0]	0.0 [0.0–1.0]^ab^	0.0 [0.0–1.0]^ab^	29.856	< 0.001
Biochemical indicators on day 1 of admission						
ALB (g/L)	35.0 [32.0–41.5]	38.0 [35.0–44.0]	41.0 [38.0–42.0]	43.0 [42.0–45.0]^abc^	27.459	< 0.001
TLC (10^9^/L)	1.0 [0.6–1.42]	0.9 [0.7–1.4]	1.4 [1.1–1.9]^ab^	1.7 [1.5–2.0]^ab^	28.265	< 0.001
RDW (%)	14.8 [13.4–15.8]	13.4 [12.6–14.3]	13 [12.5–13.6]^a^	12.8 [12.4–13.5]^a^	21.778	< 0.001
PNI (g/L + 10^9^/L)	40.0 [37.8–46.9]	44.1 [40.5–48.7]	47.2 [44.5–50.9]^a^	52 [49.6–53.6]^abc^	39.758	< 0.001
HALP (g/L)^2^	16.0 [8.6–31.4]	26.0 [14.9–42.9]	34.5 [26.7–50.0]^ab^	47.4 [36.9–59.8]^abc^	38.455	< 0.001
Prognosis						
mRS at discharge						
1–3 score	3 (15.0)	22 (48.9)	43 (70.5)^a^	24 (66.7)^a^	21.477	< 0.001
4–6 score	17 (85.0)	23 (51.1)	18 (29.5)^a^	12 (33.3)^a^		
In-hospital death						
No	8 (40.0)	40 (88.9)^a^	55 (90.2)^a^	35 (97.2)^a^	38.177	< 0.001
Yes	12 (60.0)	5 (11.1)^a^	6 (9.8)^a^	1 (2.8)^a^		
Length of hospital stay (day)	11.5 [7.5–27.5]	19.0 [11.0–26.0]	12.0 [7.0–15.0]^b^	16.0 [13.5–21.0]^c^	12.249	0.007
Total hospitalization cost (million)	4.1 [2.7–5. 5]	2.6 [1.2–6.0]	1.5 [0.8–5.5]^a^	2.4 [1.2–3.8]	9.278	0.026
Average daily hospitalization cost (million)	0.3 [0.2–0.5]	0.2 [0.1–0.3]	0.1 [0.1–0.3]^a^	0.1 [0.1–0.2]^a^	10.427	0.015

Group 1: “Low PA Rapid Decline Group”; Group 2: “Low PA Slow Decline Group”; Group 3: “Medium PA Slow Decline Group”; Group 4: “High PA Stable Group”. Bonferroni was used for multiple comparisons, "a" compared with Group 1, P < 0.05; "b" compared with Group 2, P < 0.05; "c" compared with Group 3, P < 0.05

*Alb* albumin, *BMI* body mass index, *CCI* Charlson Comorbidity Index, *HALP* Hemoglobin, Albumin, Lymphocyte and Platelet Score, *NIHSS* National Institutes of Health Stroke Scale, *PNI* Prognostic Nutritional Index Score, *RDW* red blood cell volume distribution width, *TLC* total lymphocyte count

### Random forest prediction model for early prognosis of stroke patients

**Fig. 4:**
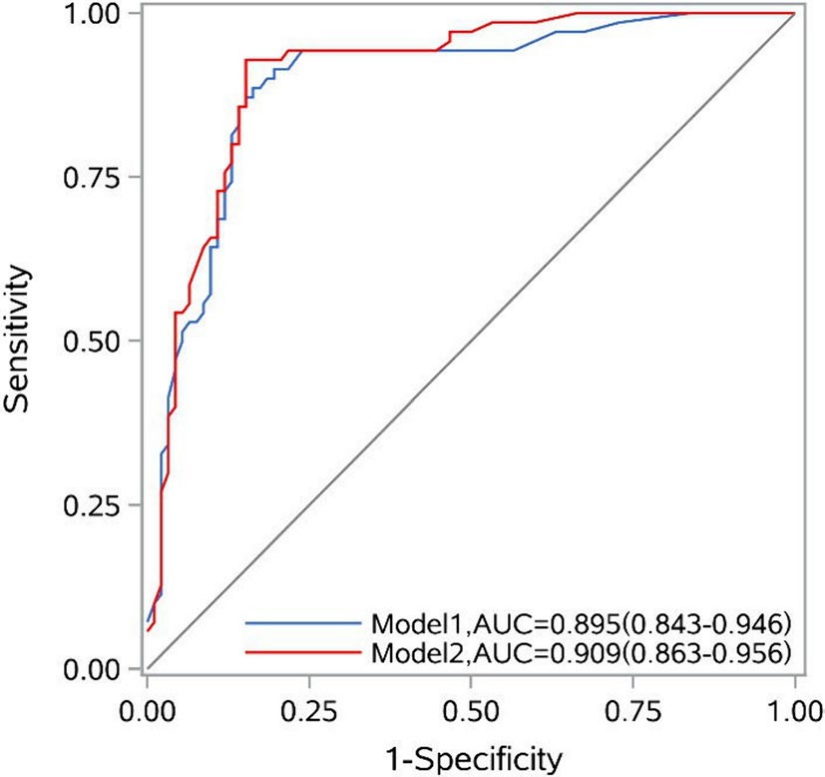
Receiver operating characteristic (ROC) curve for predicting the poor prognosis of stroke patients at hospital discharge with random forest based on tenfold cross-validation. Model 1 includes NIHSS, HALP, BMI, RDW, TLC and age. Model 2 includes the variables of Model 1 plus PA trajectory groups

Two prediction models were constructed using the random forest algorithm. Model 1 incorporated characteristic variables with predictive significance, including NIHSS, HALP, BMI, RDW, TLC and Age, while Model 2 added PA trajectory groups on the basis of Model 1. The results suggested that Model 2 had higher AUC (0.909 vs 0.895), accuracy (88.27% vs 85.80%), sensitivity (92.86% vs 88.57%), and specificity (84.78% vs 83.70%) than Model 1 (see Table 4 and Fig. 4). Compared with Model 1, Model 2 increased the diagnostic efficacy by 3.5% (IDI = 0.035, *P* < 0.001), improved the accuracy of prognostic prediction in stroke patients at hospital discharge by 17.5% (NRI = 0.175,* P* = 0.031), improved the prediction accuracy for poor prognosis in stroke patients at hospital discharge by 14.3% (NRI(+) = 0.143, *P* = 0.034), and improved the prediction accuracy for good prognosis in stroke patients at hospital discharge by 3.3% (NRI(−) = 0.033, *P* = 0.484) (see Table 5).

**Table 4:** Outcomes of different models for predicting the poor prognosis of stroke patients at hospital discharge

Model	Accuracy (%)	Sensitivity (%)	Specificity (%)	AUC (95% CI)	*P* value
Model 1	85.80	88.57	83.70	0.895 (0.843, 0.946)	< 0.001
Model 2	88.27	92.86	84.78	0.909 (0.863, 0.956)	< 0.001

Model 1 includes NIHSS, HALP, BMI, RDW, TLC and age

Model 2 includes the variables of Model 1 plus PA trajectory groups

Tenfold cross-validation results

*AUC* area under the curve, *CI* confidence interval

**Table 5:** Comparison of different prediction models for the poor prognosis of stroke patients at hospital discharge

Indicators	Value	SE	95% CI	*P* value
Difference in AUC^a^	0.014	0.007	− 0.0003, 0.029	0.055
IDI	0.035	0.008	0.019, 0.051	< 0.001
NRI	0.175	0.081	0.015, 0.175	0.031
NRI(+)	0.143	0.067	0.013, 0.143	0.034
NRI(−)	0.033	0.047	− 0.056, 0.033	0.484

*AUC* area under the curve, *CI* confidence interval, *IDI* Integrated Discrimination Improvement Index, *NRI* net reclassification improvement

^a^DeLong’s test of Model 2 vs. Model 1

## Discussion

**Fig. 5:**
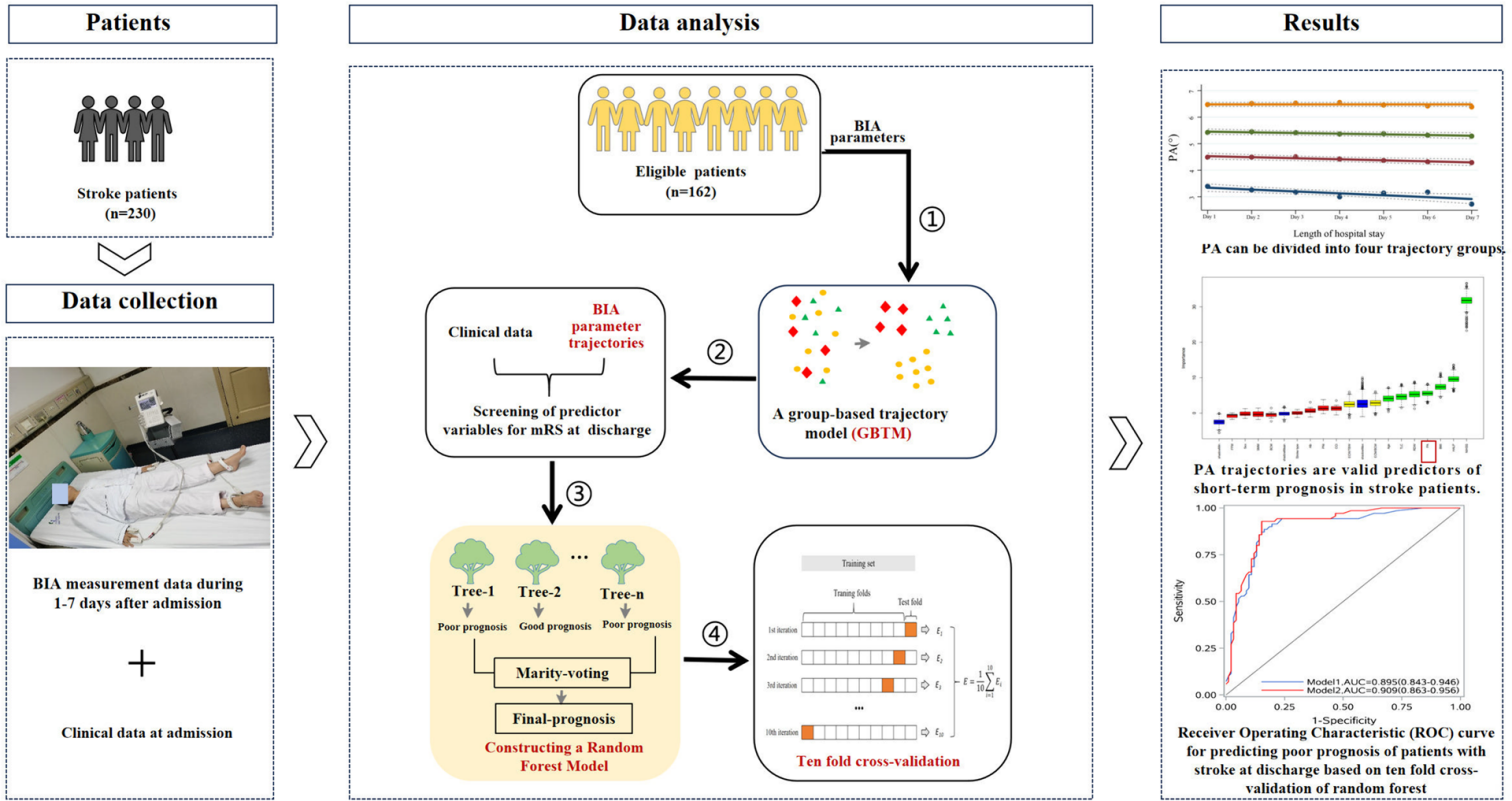
The flowchart of this study. ① Trajectory modeling using consecutively measured BIA parameters during the first 1–7 days of admission to derive optimal trajectory groupings for different BIA parameters; ② select important feature variables with predictive value, including BIA parameter trajectories and clinical indicators at admission; ③ constructing a random forest prediction model based on selected feature variables; ④ internal validation of models using ten-fold cross-validation

This is an observational study. GBTM had first been employed for the dynamic BIA parameters in 162 stroke patients during their first 1–7 days of admission, to identify the trajectories of BIA parameters that can reflect acute-phase inflammation and nutritional status and with prognostic prediction value. By combining with the clinical indicators at the time of admission, we constructed the random forest model to predict the short-term prognosis of stroke patients. Our study found that PA trajectories from BIA parameters, which represent the changes in inflammation and nutritional status during the acute phase. PA trajectories were valid predictive indicators for short-term prognosis in stroke patients. The random forest model, which simultaneously includes PA trajectories, NIHSS, HALP, BMI, RDW, TLC and age, can comprehensively reflect the disease severity at admission and the dynamic changes in acute-phase inflammation and nutritional status in stroke patients. This model has high predictive value, providing a more comprehensive and accurate reference basis for patients’ observation and prognostic prediction (see Fig. 5).

PA provides important information about cell health [33]. In disease states, the decline of PA is mainly caused by inflammation, malnutrition, or a combination of both [42]. Several studies have shown that PA is a valid predictive indicator of poor clinical outcomes in stroke patients at discharge [43–45]. However, the PA thresholds used to define poor outcomes in those studies were all static results at a single time point, leading to significant heterogeneity and limited clinical applicability. In contrast, our study collected dynamic data of PA through continuous BIA measurement, providing a more comprehensive observation of the dynamic changes and development trends in inflammation and nutritional status during the acute phase of stroke patients. In this study, patients in the “Low PA Slow Decline Group” and “Low PA Rapid Decline Group” both had lower PA at admission. This result suggests that these patients may have been in a status of malnutrition and immune impairment before admission, resulting in decreased muscle mass and reduced cellular function [43, 46]. Moreover, the lower the initial PA value is, the faster its decline over time (within 1–7 days of admission) goes, and the higher the risk of poor prognosis exists. This result indicates that patients with lower PA at admission have poorer nutritional immune status, weaker anti-stress reserve capacity, whereas the acute-phase inflammatory response and oxidative stress lead to further damage to the cellular structure and membrane integrity of the affected organs, accelerating the cell death, which manifests as a rapid decrease in PA. Therefore, low PA at admission and its decline rate are positively correlated with the overall condition and degree of organ involvement, thus affecting patient prognosis [47–49]. However, we also observed that stroke patients from the “High PA Stable Group” and the “Medium PA Slow Decline Group” had relatively better short-term prognosis with lower risks of poor clinical outcomes at discharge. Interestingly, both the “High PA Stable Group” and the “Low PA Rapid Decline Group” had longer hospital stays (11.5 [7.5–27.5] vs 16.0 [13.5–21.0] days) (see Table 3), but the short-term prognosis of patients in the two groups were totally different. These results suggest that despite these patients had all experienced the disease progression during the acute phase of stroke, patients with stronger stress recovery capabilities, due to good initial nutritional immune status, may have a better prognosis or benefit more from aggressive treatment. Therefore, PA trajectories can reflect the dynamic changes in nutritional immune status of stroke patients at admission and during the acute phase and can serve as valid indicators for predicting the early prognosis of this group of patients.

NIHSS is a classic prognostic prediction indicator in stroke patients, reflecting the severity of the primary brain injury, and its score is positively correlated with the risk of poor prognosis in this group of patients [50]. In the random forest model of this study, NIHSS is also an important predictive indicator for the short-term prognosis of stroke patients. However, it is worth noting that in the comparison of the characteristics of the PA trajectory groups, although the incidence of poor prognosis at discharge was significantly higher in the “Low PA Rapid Decline Group” than in the “High PA Stable Group”, there was no statistically significant difference in NIHSS scores at admission between the two groups. This result indicates that although the poor prognosis of stroke patients is closely related to the severity of primary brain injury, the dynamic development trend of inflammation and nutritional status during the acute phase, i.e., the secondary injury also contributes significantly to the occurrence of poor prognosis. Furthermore, the random forest model of this study showed that age, BMI, and biochemical indicators (HALP, RDW, and TLC) at admission were also valid predictive indicators for the short-term prognosis of stroke patients. Previous studies have shown that elderly stroke patients usually have lower BMI and higher NIHSS scores at admission, suggesting that this group of patients have poorer nutritional immune status, more severe primary brain injury, and a higher risk of progressing to severe cases with poorer prognosis [34, 51–54]. It is worth noting that most of the above predictive indicators are static data, only reflecting the disease status and the severity of the primary disease injury at the time of testing, and unable to dynamically assess the impact of the changing pathophysiology of the disease on the overall condition. However, the prognosis of stroke patients is influenced by both the severity of primary disease injury and the inflammation and nutritional status during the acute phase. Therefore, in this study, a random forest prediction model was built to predict the early prognosis of stroke patients by combining the PA trajectory groups and the static indicators (NIHSS, age, BMI, HALP, RDW, TLC) at admission. Its AUC was as high as 0.909, and its accuracy, sensitivity and specificity were 88.27%, 92.86% and 84.78%, respectively. The inclusion of PA trajectory groups increased the diagnostic efficacy of the model and improved the discriminative ability for poor and good prognosis in stroke patients at hospital discharge. BIA can be used as an effective assessment tool for predicting the short-term prognosis of stroke patients in the clinical environment.

## Limitations

Our study had certain limitations. First, this study is a single-center observational study, and the stroke prognostic prediction model in this study needs to be externally validated in a multi-center, large-sample population to further test the reliability of this prediction model. Second, this study only monitored the trajectory of BIA parameters and their impact on prognosis in stroke patients during their acute phase within 1–7 days of admission. It did not conduct further analysis on the impact of changes in BIA-related parameters on prognosis after 7 days of admission. Therefore, the following studies could include BIA-related parameters after 7 days of admission to better reflect the complete pathological changes of inflammation and nutritional status and their impact on prognosis. Finally, this study only focused on the short-term prognosis of stroke patients at discharge. Longer follow-up is needed in the future. Further studies are necessary to investigate the relationship between BIA parameters reflecting inflammation and nutritional status and the long-term prognosis of stroke patients.

## Conclusion

PA trajectories during the acute phase (1–7 days after admission) are valid indicators dynamically reflecting the inflammation and nutritional status in stroke patients and predicting their short-term prognosis. The random forest prediction model being built by combining the clinical indicators at admission and PA trajectories at acute phase can accurately predict the neurological function prognosis of these patients at discharge. This study has developed a new, non-invasive and continuously observable bedside assessment tool for acute-phase observation in stroke patients, especially the early identification and dynamic changes of the inflammation and nutritional status. This tool is easy to operate, quick to measure, and provides objective and accurate results, making it easily applicable in clinical settings. It is worth noting that this study also proposes that evaluating early prognosis in stroke patients should not only consider the severity of primary brain injury but also the influence of dynamic changes in acute-phase inflammation and nutritional status, i.e., secondary injury on prognosis. Adding monitoring of acute-phase disease progression in stroke patients is an innovative attempt to improve the process of clinical observation and early prognostic prediction, providing further reference for precision medical practices.

### Supplementary Information

Additional file 1. (1) BIA measurement. (2) Definition of study indicators.

Additional file 2: Table S1. The fit of the trajectory analysis model for different BIA parameters subgroups. Table S2. Trend analysis of changes over time of PA trajectory groups.

## Data Availability

The data are available from the corresponding author upon reasonable request.

## Abbreviations

Alb: Albumin
AIC: Akaike Information Criterion
AUC: Area under the receiver operating characteristic curve
AvePP: Average post hoc grouping probability
BCM: Body cell mass
BIA: Bioimpedance analysis
BIC: Bayesian information criterion
BMI: Body mass index
CCI: Charlson Comorbidity Index
CI: Confidence interval
DAMPs: Damage-associated molecular patterns
ECM: Extracellular mass
ECW: Extracellular water
FFM: Fat-free mass
GBTM: Group-based trajectory model
HALP: Hemoglobin, Albumin, Lymphocyte and Platelet Score
IDI: Integrated discrimination improvement index
mRS: Modified Rankin Scale
ML: Machine learning
NPV: Negative predictive
NRI: Net reclassification improvement
NIHSS: National Institutes of Health Stroke Scale
PA: Phase angle
PNI: Prognostic Nutritional Index Score
PPV: Positive predictive value
RDW: Red blood cell volume distribution width
ROC: Receiver operating characteristic
RF: Random forest
SMM: Skeletal muscle mass
TBW: Total body water
TLC: Total lymphocyte count
