# Supplementary material for: Predictive value of the random forest model based on bioelectrical impedance analysis parameter trajectories for short-term prognosis in stroke patients
Source: Eur J Med Res. 2024 Jul 24;29:382. doi: 10.1186/s40001-024-01964-8 (PMC11267791; doi:10.1186/s40001-024-01964-8)
Supplement: Supplementary file 1 — Additional file 1. (1) BIA measurement. (2) Definition of study indicators. [file 40001_2024_1964_MOESM1_ESM.docx]

**Supplementary Methods**

**1. BIA measurement**

The BIA measurements were performed within 7 days after admission to the NICU using a InBody S10 device(InBody Co, Ltd., Seoul, Korea) at frequencies of 1, 5, 50, 250, 500 and 1000kHz. In accordance with the manufacturer's instructions, a tetrapolar wrist-to-ankle method was used, in which two electrodes were placed approximately 5 mm apart on the dorsal surface of the right wrist and ipsilateral ankle under a supine position with arms and legs in abduction to avoid contacts with the trunk. The input variables included the patients' age, sex, height and actual body weight. The output parameters included phase angle (PhA, 50 kHz), skeletal muscle mass(SMM), fat-free mass(FFM), body cell mass(BCM), extracellular water (ECW), and total body water (TBW). SMM, FFM, BCM, ECW and TBW were calculated based on a built-in undisclosed proprietary equation developed by the manufacturer. All measurements were performed by a trained investigator and supervised by a senior investigator. We performed serial BIA measurements for each patient included in the study from 6:00 AM to 8:00 AM within 1-7 days after their admission, prior to relevant treatments such as food intake and infusion, at ambient temperature and relative humidity of 22-23℃ and 50-60%, respectively.

**2.** **Definition of study indicators**

**Body mass index(BMI):** BMI=weight/height^2^(kg/m^2^). BMI was divided into six categories on the basis of the National Institutes of Health and the World Health Organization criteria for BMI: underweight (<18.5kg/m^2^),normal weight (18.5-24.9kg/m^2^), overweight (25.0-29.9kg/m^2^), obesity (30.0-34.9kg/m^2^), serious obesity (35.0-39.9kg/m^2^), and morbid obesity (> 40.0kg/m^2^)^[1]^.

**National Institutes of Health stroke scale（NIHSS）：**The NIHSS score is the most commonly used scale for assessing neurologic function in acute stroke patients and can effectively predict the prognosis of stroke patients. The scale contains 11 dimensions, which are level of consciousness, eye movements, visual fields, facial muscle function, bilateral upper extremity activity, bilateral lower extremity activity, sensory function，

coordination (ataxia),language (aphasia),speech (dysarthria) and hemi-inattention (neglect)，and Each dimension is scored on a scale from 0 to 2, 0 to 3, or 0 to 4. The total NIHSS score ranges from 0-42, with higher scores indicating more severe neurologic impairment^[2]^.

**Charlson Comorbidity Index(CCI):** CCI is the clinimetric assessment of prognostic comorbidity, which is a broader concept than diagnostic or treatment comorbidity, and specifically on chronic conditions that impact on survival outcomes, especially long-term survival. CCI consisted of 19 items corresponding to different medical comorbid conditions. The total score of the CCI consists in a simple sum of the weights. Its score ranges from 0 to 37, with higher scores indicating not only a greater mortality risk but also more severe comorbid conditions.The assessment value within 24 hours after admission was used^[3]^.

**Albumin(Alb):** Human serum albumin, the most abundant protein in plasma, is a monomeric multi-domain macromolecule. Its normal range is 35-50g/L. Among them, hypoalbuminemia is defined as serum albumin<34g/L. Elevated parameters of inflammation and high nutritional risk are independently associated with hypoalbuminemia^[4]^.

**Total lymphocyte count(TLC):** Lymphocytes are cells of the adaptative immune system and common inflammatory markers. TLC refers to the absolute number of lymphocytes in the blood, and its normal range is (1.10-3.20*10^9^/L). TLC have been reported as valid and reliable markers for defining malnutrition^[5]^.

**Red blood cell volume distribution width(RDW):** Red blood cell (RBC) distribution width (RDW) is known to reflect the heterogeneity of RBC volume.The normal range of RDW in adults was 12.3±0.8%^[6]^. Increased RDW may comprehensively represent a variety of harmful biological mechanisms. Elevated RDW values have been shown to reflect malnutrition and to be a possible marker for accelerated red blood cell (RBC) destruction or ineffective RBC production^[6]^. RDW was associated with plasma markers of inflammation^[7]^.

**Prognostic nutritional index score(PNI):** PNI is calculated from serum albumin degree and total peripheral blood lymphocytes, i.e. PNI = serum albumin (g/L) + 5 X total lymphocytes 109 L. This index quantifies the nutritional and immune status. A PNI <45 indicates a higher risk of poor prognosis in patients^[8]^.

**Hemoglobin, Albumin, Lymphocyte and Platelet score(HALP):** HALP is calculated as hemoglobin (g/L) × albumin (g/L) level × lymphocyte count (/L)/platelet count (/L), which can be used to reflect the nutritional and immune status of stroke patients. HALP <36.65 indicates that patients are at higher risk of poor prognosis^[9]^.

**Phase angle(PA):** PA is the ratio of reactance (Xc) to resistance (R) obtained from BIA measurements and expressed as an angle. PA is a valid indicator of cell membrane integrity and cell function, where lower PA is associated with impaired cell structure and increased cell death^[10]^.

1. **Treatment and Rehabilitation for Stroke Patients**

**Medical treatments:** Stroke patients in this study included both ischemic and hemorrhagic strokes. On the one hand, medical treatment solutions for ischemic stroke patients primarily followed the Chinese Stroke Association Guidelines for Clinical Management of Cerebrovascular Disease: Clinical Management of Ischemic Cerebrovascular Disease, and the Guidelines for the Early Management of Patients with Acute Ischemic Stroke published by the American Heart Association/American Stroke Association in 2018^[11-13]^. Conventional therapeutic measures in the acute phase include single or dual antiplatelet therapy, anticoagulant therapy, and statin therapy of any intensity. Reperfusion therapeutic measures include intravenous thrombolysis and endovascular therapy. Intravenous thrombolysis(IVT) refers to the intravenous administration of tissue plasminogen activator(tPA) within 3-4.5 hours after the onset of ischemic stroke(preferably alteplase, 0.9mg/Kg, maximum dose of 90mg, with 10% of the total dose administered as a bolus over the initial 1 minute, followed by the remaining 90% of the medication dissolved in 100 ml of saline solution and administered as a continuous intravenous infusion within 1 hour). Endovascular therapy(EVT) refers to thrombectomy, balloon dilatation, stent implantation, or thrombectomy + balloon dilatation + stent implantation within 24 hours after the onset of ischemic stroke based on a comprehensive assessment of the patient's condition. On the other hand, hemorrhagic stroke treatment solutions mainly followed the Chinese Stroke Association Guidelines for Clinical Management of Cerebrovascular Disease: Clinical Management of Intracerebral Haemorrhage, and the Guidelines for the Management of Spontaneous Cerebral Hemorrhage published by the American Heart Association/American Stroke Association in 2015^[14, 15]^. All patients with cerebral hemorrhage in this study were treated conservatively with medications, which mainly included symptomatic supportive treatments such as tranexamic acid antifibrinolytic, mannitol, furosemide and other medications for reducing cranial pressure and controling blood pressure and blood sugar. Given that there is a specialized neurosurgery department in the hospital where this study was conducted, surgical treatments such as hematoma drainage via burr hole and craniotomy for hematoma removal were not involved in any of the patients in this study.

**Rehabilitation protocols:** conducting rehabilitation training for stroke patients following the “Chinese Stroke Association Guidelines for Clinical Management of Cerebrovascular Disease: Clinical Management of Stroke Rehabilitation”^[16]^. All stroke patients had received individualized and comprehensive early rehabilitation treatments after their conditions were stable (vital signs being stable, symptoms and signs no longer progressing) and being evaluated by a rehabilitation doctor based on their specific situations. Rehabilitation training involved the participation of rehabilitation therapists, patients, family members, nurses and other caregivers, with training activities mainly focusing on dailiy living, language, limb and swallowing functions. The main contents of conventional rehabilitation training were: passive exercise: passive stretching, adduction, abduction and other movements, along with knee flexion, hip external rotation and other movements. During passive exercises, patients were encouraged to exert force on their own, 2 times/d, 40 min each time, 5-10 repititions per set. Active exercise: once the patient's physical condition stablized, they were guided to exercise according to a set schedule. When muscle strength reached level II, sitting exercises were introduced. First, the head of the bed was raised, and the patient was assisted to sit up and slowly sit in a wheelchair. In addition, standing and walking training, as well as training in daily living skills were conducted. Patients were assisted in leaving the ward to gradually walk independently, go up and down stairs, and were encouraged to carry out basic activities such as holding a spoon with both hands to eat, dressing themselves, and controlling urination and defecation etc., to improve their ability to live independently. For language and swallowing function training: simple instruction training, oral-facial muscle pronunciation imitation training and repetition training etc. were applied according to the specific situations of patients for their impairments in listening, speaking, reading, writing, repetition and other obstacles. Pronunciation training started with single characters and single sounds, such as “you, me, he,” with each character being loudly pronounced repeatedly. Neuromuscular electrical stimulation was applied for swallowing function training, with cotton swabs dipped with ice water to stimulate the posterior pharyngeal wall, the soft palate, and the root of the tongue, while guiding the patient to practice swallowing actions.

**References**

[1] Pickkers P, de Keizer N, Dusseljee J, et al. Body mass index is associated with hospital mortality in critically ill patients: an observational cohort study[J]. Crit Care Med, 2013,41(8):1878-1883.

[2] Kasner S E. Clinical interpretation and use of stroke scales[J]. Lancet Neurol, 2006,5(7):603-612.

[3] Charlson M E, Carrozzino D, Guidi J, et al. Charlson Comorbidity Index: A Critical Review of Clinimetric Properties[J]. Psychother Psychosom, 2022,91(1):8-35.

[4] Eckart A, Struja T, Kutz A, et al. Relationship of Nutritional Status, Inflammation, and Serum Albumin Levels During Acute Illness: A Prospective Study[J]. Am J Med, 2020,133(6):713-722.

[5] Morey V M, Song Y D, Whang J S, et al. Can Serum Albumin Level and Total Lymphocyte Count be Surrogates for Malnutrition to Predict Wound Complications After Total Knee Arthroplasty?[J]. J Arthroplasty, 2016,31(6):1317-1321.

[6] Park K I, Kim K Y. Clinical evaluation of red cell volume distribution width (RDW)[J]. Yonsei Med J, 1987,28(4):282-290.

[7] Lippi G, Targher G, Montagnana M, et al. Relation between red blood cell distribution width and inflammatory biomarkers in a large cohort of unselected outpatients[J]. Arch Pathol Lab Med, 2009,133(4):628-632.

[8] Han X, Cai J, Li Y, et al. Baseline Objective Malnutritional Indices as Immune-Nutritional Predictors of Long-Term Recurrence in Patients with Acute Ischemic Stroke[J]. Nutrients, 2022,14(7).

[9] Tian M, Li Y, Wang X, et al. The Hemoglobin, Albumin, Lymphocyte, and Platelet (HALP) Score Is Associated With Poor Outcome of Acute Ischemic Stroke[J]. Front Neurol, 2020,11:610318.

[10] Baumgartner R N, Chumlea W C, Roche A F. Bioelectric impedance phase angle and body composition[J]. Am J Clin Nutr, 1988,48(1):16-23.

[11] Liu L, Chen W, Zhou H, et al. Chinese Stroke Association guidelines for clinical management of cerebrovascular disorders: executive summary and 2019 update of clinical management of ischaemic cerebrovascular diseases[J]. Stroke Vasc Neurol, 2020,5(2):159-176.

[12] Powers W J, Rabinstein A A, Ackerson T, et al. 2018 Guidelines for the Early Management of Patients With Acute Ischemic Stroke: A Guideline for Healthcare Professionals From the American Heart Association/American Stroke Association[J]. Stroke, 2018,49(3):e46-e110.

[13] Powers W J, Rabinstein A A, Ackerson T, et al. Guidelines for the Early Management of Patients With Acute Ischemic Stroke: 2019 Update to the 2018 Guidelines for the Early Management of Acute Ischemic Stroke: A Guideline for Healthcare Professionals From the American Heart Association/American Stroke Association[J]. Stroke, 2019,50(12):e344-e418.

[14] Cao Y, Yu S, Zhang Q, et al. Chinese Stroke Association guidelines for clinical management of cerebrovascular disorders: executive summary and 2019 update of clinical management of intracerebral haemorrhage[J]. Stroke Vasc Neurol, 2020,5(4):396-402.

[15] Hemphill J R, Greenberg S M, Anderson C S, et al. Guidelines for the Management of Spontaneous Intracerebral Hemorrhage: A Guideline for Healthcare Professionals From the American Heart Association/American Stroke Association[J]. Stroke, 2015,46(7):2032-2060.

[16] Zhang T, Zhao J, Li X, et al. Chinese Stroke Association guidelines for clinical management of cerebrovascular disorders: executive summary and 2019 update of clinical management of stroke rehabilitation[J]. Stroke Vasc Neurol, 2020,5(3):250-259.
