# Supplementary material for: Predictive value of the random forest model based on bioelectrical impedance analysis parameter trajectories for short-term prognosis in stroke patients
Source: Eur J Med Res. 2024 Jul 24;29:382. doi: 10.1186/s40001-024-01964-8 (PMC11267791; doi:10.1186/s40001-024-01964-8)
Supplement: Supplementary file 2 — Additional file 2: Table S1. The fit of the trajectory analysis model for different BIA parameters subgroups. Table S2. Trend analysis of changes over time of PA trajectory groups. [file 40001_2024_1964_MOESM2_ESM.docx]

**Supplementary Table**

**Table S1. The fit of the trajectory analysis model for different BIA parameters subgroups**

| **BIA parameters** | **Number of subgroups** | **LL** | **BIC** | **AIC** | **Entropy** | **Participants**  **per group，**  **N (%)** | **AVEPP** |
| --- | --- | --- | --- | --- | --- | --- | --- |
| **PA(°)** | 1 | -1486.62 | -1493.51 | -1488.62 | — | G1=162(100%) | G1=1.00 |
|  | 2 | -1146.84 | -1160.62 | -1150.84 | 0.909 | G1=65(40.12%)/  G2=97(59.88%) | G1=0.97/  G2=0.98 |
|  | 3 | -914.37 | -941.92 | -922.37 | 0.936 | G1=36(22.22%)/  G2=79(48.77%)/  G3=47(29.01%) | G1=0.97/  G2=0.98/  G3=0.96 |
|  | 4 | -779.78 | -817.67 | -790.78 | 0.941 | G1=20(12.35%)/  G2=45(27.78%)/  G3=61(37.65%)/  G4=36(22.22%) | G1=0.99/  G2=0.96/  G3=0.98/  G4=0.98 |
|  | 5 | -678.18 | -722.95 | -691.18 | 0.956 | G1=18(11.11%)/  G2=32(19.75%)/  G3=58(35.80%)/  G4=40(24.69%)/  G5=14(8.64%) | G1=0.99/  G2=0.97/  G3=0.98/  G4=0.96/  G5=0.96 |
| **SMM(kg)** | 1 | -2909.57 | -2916.46 | -2911.57 | — | G1= 162(100%) | G1=1.00 |
|  | 2 | -2429.82 | -2443.59 | -2433.82 | 0.969 | G1=63(38.89%)/  G2=99(61.11%) | G1=0.99/  G2=0.99 |
|  | 3 | -2108.56 | -2129.23 | -2114.56 | 0.964 | G1=50(30.86%)/  G2=70(43.21%)/  G3=42(25.93%) | G1=0.99/  G2=0.98/  G3=0.99 |
|  | 4 | -1989.7 | -2017.25 | -1997.7 | 0.949 | G1=44(27.16%)/  G2=29(17.90%)/  G3=54(33.33%)/  G4=35(21.60%) | G1=0.99/  G2=0.98/  G3=0.96/  G4=0.97 |
|  | 5 | -1896.73 | -1931.17 | -1906.73 | 0.960 | G1=13(8.02%)/  G2=35(21.60%)/  G3=36(22.22%)/  G4=45(27.78%)/  G5=33(20.37%) | G1=0.95/  G2=0.99/  G3=0.96/  G4=0.97/  G5=0.98 |
| **FFM(kg)** | 1 | -3381.6 | -3388.49 | -3383.6 | — | G1= 162(100%) | G1=1.00 |
|  | 2 | -2910.87 | -2924.65 | -2914.87 | 0.955 | G1=63(38.89%)/  G2=99(61.11%) | G1=0.99/  G2=0.99 |
|  | 3 | -2598.46 | -2619.13 | -2604.46 | 0.963 | G1=54(33.33%)/  G2=65(40.12%)/  G3=43(26.54%) | G1=0.99/  G2=0.99/  G3=0.99 |
|  | 4 | -2464.99 | -2492.54 | -2472.99 | 0.965 | G1=43(26.54%)/  G2=37(22.84%)/  G3=49(30.25%)/  G4=33(20.37%) | G1=0.99/  G2=0.98/  G3=0.97/  G4=0.99 |
|  | 5 | -2380.33 | -2414.76 | -2390.33 | 0.953 | G1=25(15.43%)/  G2=31(19.14%)/  G3=46(28.40%)/  G4=34(20.99%)/  G5=26(16.05%) | G1=0.97/  G2=0.97/  G3=0.98/  G4=0.96/  G5=0.98 |
| **BCM(kg)** | 1 | -2999 | -3005.88 | -3001 | — | G1= 162(100%) | G1=1.00 |
|  | 2 | -2516.68 | -2530.46 | -2520.68 | 0.969 | G1=63(38.89%)/  G2=99(61.11%) | G1=0.99/  G2=0.99 |
|  | 3 | -2195.15 | -2215.81 | -2201.15 | 0.964 | G1=50(30.86%)/  G2=70(43.21%)/  G3=42(25.93%) | G1=0.99/  G2=0.98/  G3=0.99 |
|  | 4 | -2069.55 | -2097.1 | -2077.55 | 0.952 | G1=44(27.16%)/  G2=32(19.75%)/  G3=54(33.33%)/  G4=32(19.75%) | G1=0.99/  G2=0.95/  G3=0.97/  G4=0.99 |
|  | 5 | -1975.41 | -2009.85 | -1985.41 | 0.938 | G1=23(14.20%)/  G2=27(16.67%)/  G3=41(25.31%)/  G4=42(25.93%)/  G5=29(17.90%) | G1=0.94/  G2=0.99/  G3=0.96/  G4=0.97/  G5=0.99 |
| **ECM/BCM** | 1 | 2121.6 | 2111.26 | 2118.6 | — | G1= 162(100%) | G1=1.00 |
|  | 2 | 2426.02 | 2408.8 | 2421.02 | 0.944 | G1=108(66.67%)/  G2=54(33.33%) | G1=0.99/  G2=0.96 |
|  | 3 | 2583.47 | 2559.36 | 2576.47 | 0.923 | G1=39(24.07%)/  G2=95(58.64%)/  G3=28(17.28%) | G1=0.96/  G2=0.96/  G3=0.99 |
|  | 4 | 2783.82 | 2749.39 | 2773.82 | 0.957 | G1=34(20.99%)/  G2=44(27.16%)/  G3=78(48.15%)/  G4=6(3.70%) | G1=0.98/  G2=0.98/  G3=0.97/  G4=1.00 |
|  | 5 | 2868.98 | 2827.65 | 2856.98 | 0.968 | G1=34(20.99%)/  G2=36(22.22%)/  G3=74(45.68%)/  G4=13(8.02%)/  G5=5(3.09%) | G1=0.97/  G2=0.98/  G3=0.98/  G4=0.99/  G5=0.99 |
| **ECW/TBW** | 1 | 2746.51 | 2739.65 | 2744.51 | — | G1= 162(100%) | G1=1.00 |
|  | 2 | 2946.96 | 2933.23 | 2942.96 | 0.920 | G1=120(74.07%)/  G2=42(25.93%) | G1=0.98/  G2=0.98 |
|  | 3 | 3044.66 | 3020.62 | 3037.66 | 0.875 | G1=52(32.1%)/  G2=88(54.32%)/  G3=22(13.58%) | G1=0.93/  G2=0.95/  G3=0.93 |
|  | 4 | 3097.75 | 3053.1 | 3084.75 | 0.899 | G1=34(20.99%)/  G2=87(53.7%)/  G3=36(22.22%)/  G4=5(3.09%) | G1=0.94/  G2=0.94/  G3=0.93/  G4=0.98 |
|  | 5 | 3136.39 | 3081.45 | 3120.39 | 0.908 | G1=33(20.37%)/  G2=30(18.52%)/  G3=85(52.47%)/  G4=13(8.02%)/  G5=1(0.62%) | G1=0.94/  G2=0.96/  G3=0.94/  G4=0.97/  G5=1 |

Note: Shaded areas(blue) indicate the most satisfied trajectory model of each BIA parameter.

**Table S2. Trend analysis of changes over time of PA trajectory groups**

|  | **Trajectory groups** | **parameters** | **β** | **SE** | **t value** | **p value** |  |
| --- | --- | --- | --- | --- | --- | --- | --- |
| **PA(°)** | Group 1 | Intercept | 3.412 | 0.091 | 37.392 | <0.001 | |
|  |  | Linear | -0.071 | 0.023 | -3.137 | 0.002 | |
|  | Group 2 | Intercept | 4.569 | 0.068 | 67.164 | <0.001 | |
|  |  | Linear | -0.039 | 0.014 | -2.761 | 0.006 | |
|  | Group 3 | Intercept | 5.479 | 0.059 | 92.602 | <0.001 | |
|  |  | Linear | -0.026 | 0.012 | -2.135 | 0.033 | |
|  | Group 4 | Intercept | 6.481 | 0.039 | 164.860 | <0.001 | |
